# Time‐lapse camera trapping as an alternative to pitfall trapping for estimating activity of leaf litter arthropods

**DOI:** 10.1002/ece3.3275

**Published:** 2017-08-14

**Authors:** Rachael A. Collett, Diana O. Fisher

**Affiliations:** ^1^ School of Biological Sciences The University of Queensland St Lucia QLD Australia

**Keywords:** insectivorous, insects, invertebrate sampling, prey availability, time lapse

## Abstract

Pitfall trapping is the standard technique to estimate activity and relative abundance of leaf litter arthropods. Pitfall trapping is not ideal for long‐term sampling because it is lethal, labor‐intensive, and may have taxonomic sampling biases. We test an alternative sampling method that can be left in place for several months at a time: verticallyplaced time‐lapse camera traps that have a short focal distance, enabling identification of small arthropods. We tested the effectiveness of these time‐lapse cameras, and quantified escape and avoidance behavior of arthropod orders encountering pitfall traps by placing cameras programed with a range of sampling intervals above pitfalls, to assess numerical, taxonomic, and body size differences in samples collected by the two methods. Cameras programed with 1‐ or 15‐min intervals recorded around twice as many arthropod taxa per day and a third more individuals per day than pitfall traps. Hymenoptera (ants), Embioptera (webspinners), and Blattodea (cockroaches) frequently escaped from pitfalls so were particularly under‐sampled by them. The time‐lapse camera method effectively samples litter arthropods to collect long‐term data. It is standardized, non‐lethal, and does not alter the substrate or require frequent visits.

## 1 INTRODUCTION

Pitfall trapping is the most commonly used method to estimate activity and relative abundance of ground‐dwelling arthropods in ecological studies, and for monitoring (Spence & Niemela, 1994; Lovei & Sunderland, 1996). For example, researchers have used pitfall trapping data to calculate measures of prey availability in studies of dietary ecology (Fisher & Dickman, 1993a,b) and life history evolution (Fisher, Dickman, Jones, & Blomberg, 2013), as indicators of disturbance in studies of fire and logging (Lawton et al., 1998; Barrow, Parr, & Kohen, 2007), to estimate rates of extinction (Dirzo et al., 2014), to test hypotheses in community ecology and biogeography (Dickman, 1988; Driscoll, 2005), and in agricultural pest management (Kromp, 1999). To date, there have not been any practical alternative methods for such studies (Spence & Niemela, 1994).

Pitfall trapping can be a useful method to sample arthropod availability because the traps are cheap and simple to deploy, and daily samples from each trap can be kept in preservative and used to identify the samples to species, as reference collections in dietary analysis, or to obtain genetic information (Topping & Sunderland, 1992; Santos, Cabanas, & Pereira, 2007).

Pitfall trapping also has a number of logistical drawbacks. It can be labor‐intensive as pitfall traps must be emptied and refilled with preservative frequently to obtain replicates, and to ensure that they are still sampling effectively (e.g., Parker, Skinner, & Gouli, 1997; Fisher & Dickman, 1993a; McKinnon, Picotin, Buldoc, Juilett, & Bety, 2012). If left unmonitored, pitfall traps can flood, be dug out of the ground and destroyed by animals, or otherwise disturbed. Sampling of remote areas is thus limited to relatively short periods during field trips. Pitfall traps can also by‐catch small vertebrates, which means that their use is increasingly restricted by animal ethics committees (Lange, Gossner, & Weisser, 2011). Digging in pitfall traps is unacceptable at some sites such as land of special significance to indigenous owners or environmentally sensitive areas, and too difficult in some substrates such as rock pavement.

A large methodological issue with using pitfall traps is that they do not sample taxa at random from the leaf litter arthropod community (Luff, 1975; Baars, 1979; Topping & Luff, 1995). For example, Luff (1975) found that large Coleoptera (beetle) individuals were not efficiently caught by pitfall traps, and that escape rates were high. Baars (1979) compared two species of carabid beetle and found that one was eight times more likely to be trapped than the other. Pitfall trap captures can vary with trap design, preservative type, and surrounding substrate (Spence & Niemela, 1994; Melbourne, 1999; Pekar, 2002; Schmidt, Clough, Schulz, Westphalen, & Tscharntke, 2006) and soil disturbance can lead to more individuals of particular taxa being sampled immediately after traps are set (Greenslade, 1973; Schirmel, Lenze, Katzmann, & Buchholz, 2010). Until now, the magnitude and direction of capture bias has not been studied in natural environments, and in whole communities of arthropods.

A large proportion of the world's vertebrate fauna is insectivorous. Many birds and mammals, and most lizards and frogs eat arthropod prey. The prey base for these animals appears to be declining globally (Dirzo et al., 2014). Long‐term, repeatable studies of arthropod community composition and relative abundance will allow us to better detect and act on declines. However, published studies vary in their pitfall trapping methods. Variation between studies in the number of traps, configuration, preservative type, and amount, and failure to report exact protocols means that comparison of relative abundance of arthropods between published studies and between past and present is problematic. Here, we present a new method to assess arthropod relative abundance, activity, and community composition using time‐lapse camera traps. We use the Reconyx PC850 model, customized with a short focal distance (250 mm) (Soininen, Jensvoll, & Ims, 2013) so that tiny arthropods are in sharp focus. Camera traps are now commonplace for monitoring vertebrates (Meek et al., 2014; De Bondi et al. 2010; Vine et al., 2009), but have not previously been used to collect extensive field data on litter arthropods.

The major aim of this study is to test the effectiveness of camera trapping versus pitfall trapping to sample litter arthropods, specifically:

1. To determine whether there is a difference in the number of arthropod taxa sampled by pitfall and camera traps.
2. To determine whether there is a difference in the mean body length of arthropods sampled by pitfall and camera traps.
3. To quantify the differential escape behavior of arthropod taxa in the wild using time‐lapse camera traps placed above pitfall traps.

We quantify captures using cameras programed with four recording intervals in Australian rainforest and sclerophyll (Eucalypt) forest between 2012 and 2015.

## 2 METHODS

### 2.1 Sampling and measurement of arthropods

We used five sites at two locations in south‐east Queensland, Australia: site one was in upland rainforest at Springbrook National Park (−28.23ºS, 153.28ºE, 900 m asl) and sites two to five were at Conondale National Park (−26.55°S, 152.44°E, 100–800 m asl), including lowland and upland rainforest and sclerophyll forest. We sampled at Springbrook for 3 weeks, in spring and summer 2012 and summer 2014. We sampled at Conondale National Park for 16 weeks in winter and spring 2015.

At each site, we buried five pitfall traps (200 ml white plastic cups with a diameter of 70 mm) flush with the ground at 20 m intervals and half‐filled them with 70% ethanol. We chose to use plastic cups because they (or similar plastic containers) are widely used for arthropod pitfall trapping (e.g., Fisher & Dickman, 1993a,b; Driscoll, 2005). Traps were checked at dawn every morning. Each day, we stored arthropods from pitfall traps in individual specimen jars containing 70% ethanol for identification to order, and body length measurement in the laboratory.

We positioned 17 cameras vertically (with the lens and camera facing the ground) (Meek et al., 2014; Rovero, Zimmerman, Berzi, & Meek, 2013), 250 mm above the ground (the fixed focal distance), on frames attached to trees. Cameras photographed individual pitfall traps and the surrounding field of view (200 × 150 mm) for seven to eight consecutive days at Springbrook, and 14 consecutive days at Conondale National Park at each pitfall location. The field of view of cameras was 0.03 m^2^ (30,000 mm^2^), around eight times as large as the area of the pitfall trap. In 2012, we programed cameras to take a photograph once every 15, 30, or 60 s (with three replicates for each time interval) between 4 p.m. and 6 a.m. In 2014, we programed cameras to take three pictures on rapidfire with a 15‐min interval between picture sets for 24 hr. In 2015, we programed all of the cameras to take three pictures on rapidfire with a 15‐min interval between picture sets between 4 p.m. and 6 a.m. Thus, there were five treatments: cameras with 15‐s interval (*n* = 7 camera locations), cameras with 30‐s interval (*n* = 2 camera locations), cameras with 60‐s interval (*n* = 4 camera locations), cameras with 900‐s interval (*n* = 11 camera locations), and pitfall trap lines at cameras that were emptied every 24 hr (*n* = 24 locations). The total number of trap nights at all sites was 125 for the cameras and 555 for the pitfalls.

Under each camera, we placed a clear plastic or wooden ruler on one side of the field of view in order to measure the body length of individual arthropods in photographs. Although the cameras are waterproof, to prevent water pooling and entering the cases in torrential rain, we fashioned rain covers out of 25 cm × 18 cm plastic containers.

Published studies commonly report relative abundance of arthropods in broad categories of order or body size (e.g., Fisher & Dickman, 1993a; Douglas, Vickery, & Benton, 2010). We identified photographed and captured arthropods to the taxonomic level of order in most cases. We classified larvae (Coleopteran and Lepidopteran), Oligochaetes, and Opiliones in these separate categories. We refer to all categories as “orders” or “taxa.” Only arthropods and larvae larger than 1 mm were included in the analysis because this was the minimum size for accurate identification to order from photographs. However, we still recorded the presence of arthropods smaller than 1 mm in the datasheet if we found them in camera or pitfall trap samples. In most cases, identification to order could be made in 5 s. Sequential time‐lapse photographs are nearly identical, so we could quickly discard photographs without arthropods. On average, it took us 1 min to analyze 1 day of photos recorded using the 15‐min time‐lapse interval.

We classified an arthropod sighting as an “escape” if successive time‐lapse photographs showed the arthropod walking over the edge of a pitfall trap and then back out. A sighting was classified as an “avoidance,” if an arthropod walked to the rim of a pitfall trap and then changed direction, preventing it from falling in. If the same arthropod appeared in consecutive time‐lapse photographs, it was only recorded once.

### 2.2 Statistical analysis of arthropods

We used a mixed‐effects model with a Poisson distribution to test whether the number of taxa sampled per location per day differed between cameras and pitfalls, with daily trap identity as a random factor (in the R package MASS, Venables & Ripley, 2002). To test whether there was a difference in the mean body length of arthropods sampled by cameras and pitfall traps, we used linear mixed‐effects models with daily trap identity as a random factor. We used a Chi‐squared test to find whether the proportion of escapes and avoidances of pitfall traps varied between arthropod taxa.

## 3 RESULTS

**Figure 1:**
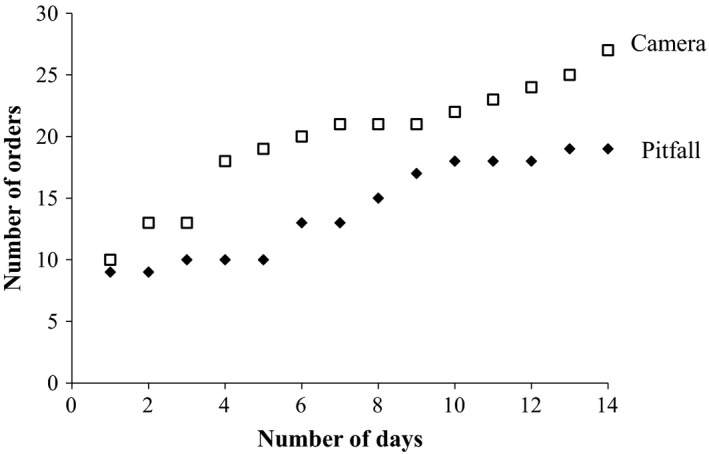
The cumulative number of arthropod orders detected per day by pitfall and camera traps (15‐, 30‐, 60‐s and 15‐min time intervals pooled)

**Figure 2:**
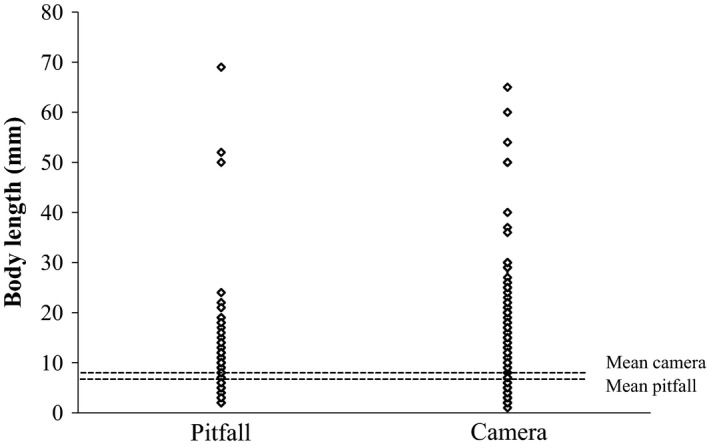
The distribution and mean body length of arthropods captured by pitfall and camera traps (15‐, 30‐, 60‐s and 15‐min time intervals pooled)

When the camera sampling intervals were combined, cameras recorded 37% more taxa than did pitfall traps at the same locations and times; cameras recorded a mean of 6.8 ± 0.56 (SEM) orders per day and pitfalls 4.29 ± 0.41 (*t* = −4.53 _15,48_, *p* < .001). In 2‐week sampling periods, the cumulative number of taxa sampled by cameras outstripped the number sampled by pitfall traps from the first day (Figure 1). The mean number of individuals encountered per location per day, including all camera time intervals, was 14.05 ± 2.67 (SEM) for cameras and 9.67 ± 1.76 for pitfalls. When we compared the number of orders sampled per day in the five treatments (cameras with 15‐s intervals, cameras with 30‐s intervals, cameras with 60‐s intervals, cameras with 900‐s intervals, and pitfall traps at cameras) in a mixed‐effects model, pitfall traps sampled around half the number of orders per day (4.3 + 0.4 orders) than cameras with 900‐s intervals (7.7 + 1, *t* = 4.1 _3,29_, *p* = .0003) and 60‐s intervals (7.8 + 0.8, *t* = 4.2 _3,29_, *p* = .0002), but not significantly fewer than the limited number of cameras with 30‐ (6.5 + 1.5, *t* = 1.7 _3,29_, *p* = .10) or 15‐s intervals (5 + 0.6, *t* = 1.3 _3,29_, *p* = .21). The mean body length of arthropods sampled did not differ between cameras and pitfall traps (Figure 2, *t* = −1.28 _25,2952_, *p* = .20) (Appendix 1).

**Figure 3:**
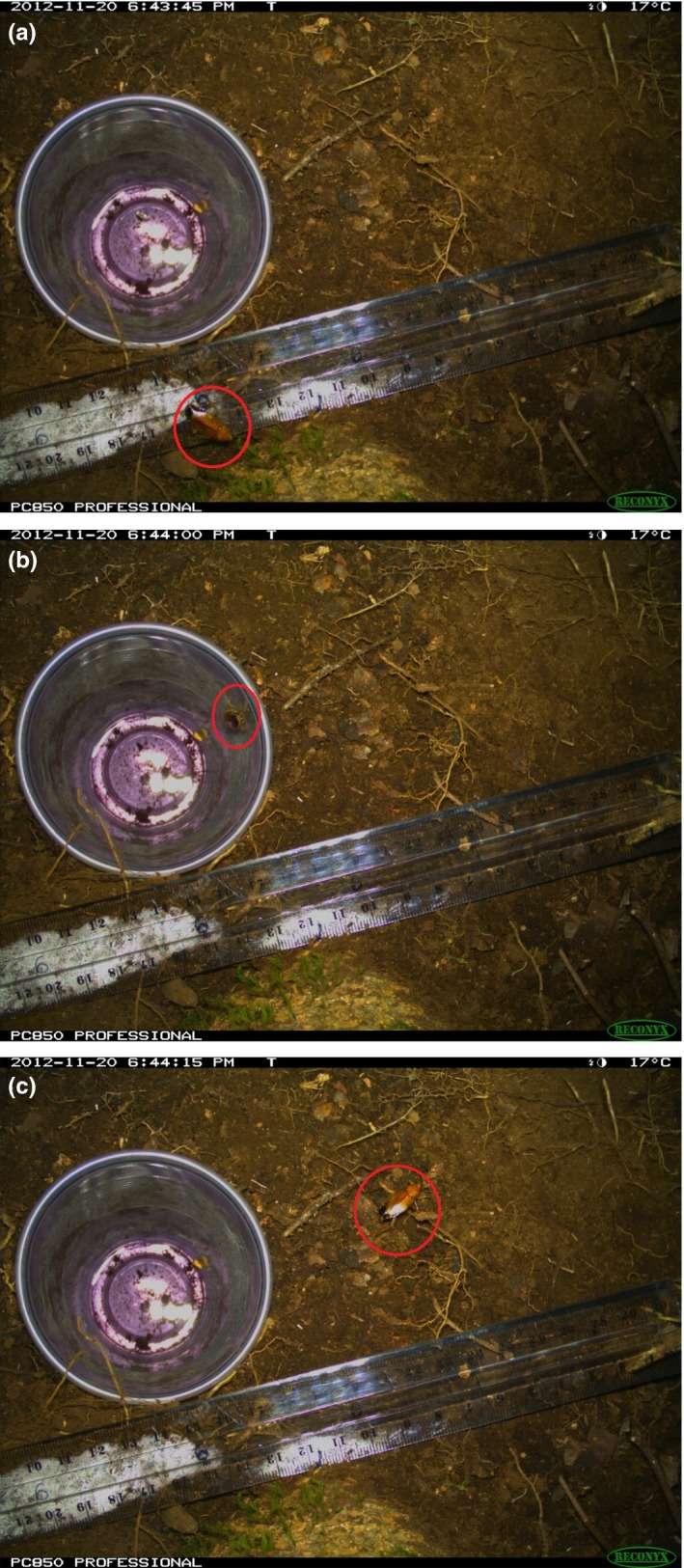
Three consecutive photographs from a camera trap, showing a cockroach escaping a pitfall trap

The camera trap record of escapes and avoidances showed that a quarter of the photographed arthropods that approached pitfall traps did not fall into the fluid, and sampling by pitfall traps was taxonomically biased (Chi‐squared test, χ^2^ = 916, *df* = 4, *p* ≪ .0001). Ants (Hymenoptera) escaped or avoided traps 25% of the time, webspinners (Embioptera) 31%, and cockroaches (Blattodea) 46%. Spiders (Araneae) were occasionally (12% of the time) able to escape, but were never seen avoiding pitfall traps (Figure 3). Other taxa did not avoid or escape from pitfalls (Table 1).

**Table 1:** Number of escapes from and avoidances of pitfall traps for each major arthropod order detected by cameras (15‐, 30‐, and 60‐s intervals pooled) placed over pitfall traps

Order	Escapes and avoidances/total captures (for pitfall traps)
Blattodea	6/13
Hymenoptera	234/920
Embioptera	130/419
Araneae	2/17
Coleoptera	0/22
Orthoptera	0/14
Hemiptera	0/16
Chilopoda	0/11
Diptera	0/6
Amphipoda	0/6
Spirobolida	0/6
Larvae (Coleoptera and Lepidoptera pooled)	0/2
Thysanoptera	0/1
Stylommatophora	0/1
Phasmatodea	0/1
Opiliones	0/1

Although the proportion of time when photographs were actually being taken each day was small (less than 2% of the day), examination of successive photographs showed that individual arthropods were typically at risk of recording or capture for periods of time that included multiple photographs. For example, in the 15‐min interval samples, individual arthropods (assumed to be the same individual based on appearance and position in successive frames) stayed in the frame for a mean time of 32 ± 13 min, and only 5% of arthropods appeared in only one frame. One caterpillar took 4 hr to traverse the frame, and a spider remained in view (apparently hunting in a “sit‐and‐wait” posture) for 15.5 hr.

## 4 DISCUSSION

### 4.1 Detection of taxa

Since the 1980s, researchers have sought ways to collect mid‐ to long‐term, time‐stamped, standardized, and replicated data on relative abundance of terrestrial arthropod taxa. Our new camera method is a simple and effective way to sample relative abundance and activity of leaf litter arthropods. The cameras can provide time‐stamped data, to the taxonomic level of order, over large temporal and spatial scales. Our cameras detected the presence of leaf litter arthropod orders more quickly than pitfall traps and were just as efficient at detecting the range of arthropod body sizes in rainforest and sclerophyll forest.

It is likely that more taxa were detected by the cameras than the pitfalls because more individuals were sampled by the cameras, increasing the chance of observing rare taxa. Cameras captured 37% more individuals than pitfalls per day, probably because the sample area is around eight times larger and cameras recorded many individuals that did not fall into the pitfall traps (26% of individuals photographed approaching pitfall traps avoided or escaped being trapped).

Arthropods are at risk of capture in pitfall traps 24 hr a day, but can only be photographed by time‐lapse camera traps for a fraction of this time. This did not decrease the effectiveness of the cameras, as we found that arthropods remained in the field of view for more than 30 min on average. A time‐lapse schedule set to take a photograph every 15 min for a week or more was as effective for detecting arthropod orders as a regime of daily pitfall trapping. There was no advantage in very short (15 or 30 s) intervals between pictures. Using a 15‐min time‐lapse interval gives a battery life of up to 4 months, resulting in a tractable (although large) number of photographs to analyze.

### 4.2 Inferences concerning population abundance and long‐term sampling

Instead of using pitfall traps, arthropod densities and body size distributions are sometimes calculated using leaf litter heat extraction, suction sampling, or fenced photoeclectors, as it is assumed these techniques can census all individuals in an area (Lang, 2000; Spence & Niemela, 1994; Holland & Smith 1999; Zhao et al. 2013). This assumption is untrue because large arthropods have been shown to flee more effectively when approached, and as a result these methods sample smaller mean body sizes than pitfall traps (Spence & Niemela, 1994; Lang, 2000). No current method can measure absolute abundance because the number of undetected individuals is unknown, unlike in mark‐recapture or distance sampling of larger fauna (Pollock, Nichols, Brownie, & Hines, 1990; Buckland, Anderson, & Laake, 1993). Similarly, the camera method presented here cannot estimate absolute abundance. However, camera trapping does not deplete local arthropod populations over time. This means that camera traps can provide more accurate data about long‐term arthropod activity in an area than lethal trapping. Schirmel et al. (2010) showed that for most arthropods, capture rate in pitfall traps decreased with longer sampling intervals, suggesting that repeated pitfall trapping depleted local populations.

Long‐term sampling methods should be able to separate samples into short time intervals because long‐term cumulative samples are inadequate for most ecological questions. Some time‐sorting pitfall traps have been developed to collect daily samples remotely, but these have had limited and very specific applications. For example, Shuman, Coffelt, and Weaver (1996) developed a time‐stamped pitfall method for monitoring increases in grain pests in indoor silos, and a rotating apparatus programed to sample day and night separately has received some use (Chapman & Armstrong, 1997; Kliewe, 1998; Buchholz, 2009). These field methods have not been widely adopted by ecologists because they are limited to small areas for short time periods, are not commercially available, require mechanical and electronic expertise to construct, and are labor‐intensive in comparison with pitfall traps. The camera trap time‐lapse sampling method overcomes these issues.

### 4.3 Differential escape behavior of arthropod taxa in the wild

Our analysis of the behavior of arthropods encountering pitfall traps is the first to show the direction of taxonomic sampling bias in a natural environment, comparing multiple orders of arthropods. Ants, cockroaches, webspinners, and spiders were under‐sampled by conventional pitfall trapping. The ability of different arthropod taxa to escape pitfall traps seems to be related to behavior and style of locomotion. Beetles blundered over the edge of pitfall traps, whereas ants, spiders, and cockroaches were able to climb up and down the walls and sometimes avoided the preserving fluid. Cockroaches and ants have specialized tarsi enabling adhesion to smooth surfaces and long antennae which help them to detect the rim of a pitfall trap and retreat (Halsall & Wratten, 1988; Arnold, 1974). In one case, a spider was not at risk of capture because it remained motionless in an apparent sit‐and‐wait hunting posture throughout most of the sampling day. Embioptera were abundant in our camera trap samples with concurrent pitfall trapping, but it is unusual to see them on the soil surface (Ross, 2000). Soil disruption associated with pitfall trapping may have unnaturally increased their surface activity (Digweed 1995). Crickets observed on camera never escaped pitfall traps because they jumped directly into the preserving fluid. Sperber, Soares, and Pereira (2007) have shown that crickets can be over‐sampled in short‐term pitfall traps because vibration from human activity causes a leaping response.

Although the time‐lapse camera method solves the pitfall trapping bias of taxonomic differences in arthropod ability to escape and avoid traps, similarly to pitfall trapping (and all other available arthropod sampling methods), camera traps cannot accurately measure absolute abundance. The camera method quantifies arthropod availability (a combination of activity and abundance) because the likelihood of capturing an arthropod will be influenced by movement rate, behavior, and locomotion. Certain arthropod orders are likely to remain in the frame for longer periods of time, increasing their chances of being detected.

### 4.4 Applicability of camera trapping

Time‐lapse camera traps can be used to replace or complement pitfall trapping to sample leaf litter arthropods for many different types of ecological studies or monitoring scenarioes. For example, they can be used in studies of: 1) prey availability (e.g., Fisher & Dickman, 1993a; Dickman, 1988) 2) arthropod behavior—looking at interactions between individuals (e.g., Machado & Raimundo, 2001) or monitoring growth and activity 3) community ecology—for example looking at the zonation of arthropods in time and space (e.g., Jaramillo, Contreras, & Duarte, 2003), or 4) environmental disturbance—such as looking at the effect of fire on arthropod populations (e.g., Collett & Neumann, 1995). We are currently using camera traps to look at large‐scale biogeographical patterns of arthropod seasonality and availability. We propose that time‐lapse cameras are particularly suitable for studies of prey availability because they sample in the same way that a predator encounters prey. For example, the cameras are more likely to capture a slow moving caterpillar than a cricket, but a predator would also be more likely to encounter and capture the caterpillar.

Limitations of using camera trap sampling are that leaf litter arthropods can only accurately be identified to order, tiny arthropods cannot be identified, and physical specimens are not collected. This means that camera trapping is not suitable for studies requiring species identification, genetic samples, or focusing on very small species. Additionally, changing conditions may make arthropods more difficult to identify the longer the cameras are left in the field. For example, fallen branches or soaking rainfall may make it difficult to detect arthropods.

## 5 CONCLUSION

Camera traps with programmable time‐lapse recording and short focal distance are suitable for ecological studies and monitoring of leaf litter arthropods. Cameras can solve biases associated with pitfall trapping, including differing escape abilities of arthropod taxa and provide a standardized, long‐term sampling method.

## AUTHOR'S CONTRIBUTIONS

R.C. and D.F. conducted the field work. R.C. performed the lab work (arthropod identification). R.C. and D.F. did the statistical analysis and contributed equally to the manuscript.

## Figures and Tables

**Figure A1:**
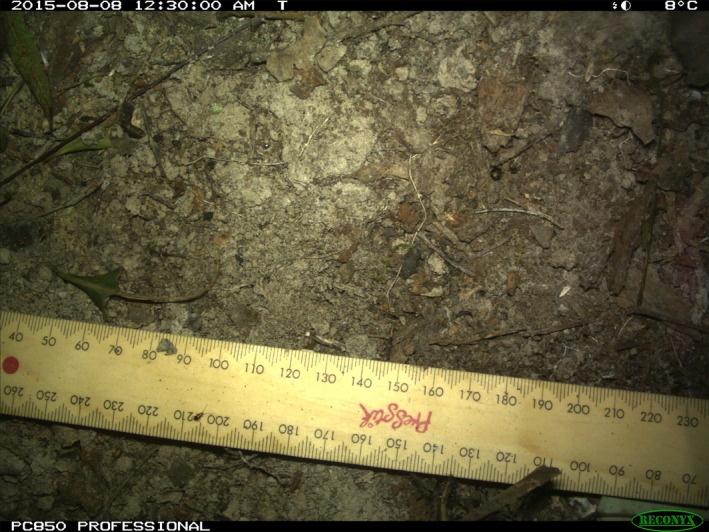
2 mm Blattodea

**Figure A2:**
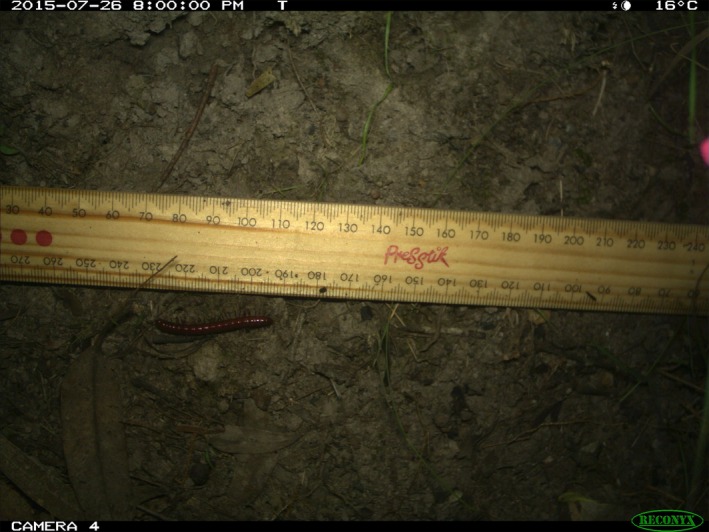
35 mm Spirobolida
